# Acid Catalyzed Alcoholysis of Sulfinamides: Unusual Stereochemistry, Kinetics and a Question of Mechanism Involving Sulfurane Intermediates and Their Pseudorotation

**DOI:** 10.3390/molecules20022949

**Published:** 2015-02-11

**Authors:** Bogdan Bujnicki, Józef Drabowicz, Marian Mikołajczyk

**Affiliations:** Department of Heteroorganic Chemistry, Centre of Molecular and Macromolecular Studies, Polish Academy of Sciences, Sienkiewicza 112, 90-363 Łódź, Poland; E-Mails: bogbujni@cbmm.lodz.pl (B.B.); draj@cbmm.lodz.pl (J.D.)

**Keywords:** sulfur chirality, stereoselective synthesis, chiral sulfinamides, chiral sulfinates, enantiomer interconversion, nucleophilic substitution, addition-elimination mechanism, sulfuranes, pseudorotation

## Abstract

The synthesis of optically active sulfinic acid esters has been accomplished by the acid catalyzed alcoholysis of optically active sulfinamides. Sulfinates are formed in this reaction with a full or predominant inversion of configuration at chiral sulfur or with predominant retention of configuration. The steric course of the reaction depends mainly on the size of the dialkylamido group in the sulfinamides and of the alcohols used as nucleophilic reagents. It has been found that bulky reaction components preferentially form sulfinates with retention of configuration. It has been demonstrated that the stereochemical outcome of the reaction can be changed from inversion to retention and *vice versa* by adding inorganic salts to the acidic reaction medium. The unusual stereochemistry of this typical bimolecular nucleophilic substitution reaction, as confirmed by kinetic measurements, has been rationalized in terms of the addition-elimination mechanism, A-E, involving sulfuranes as intermediates which undergo pseudorotations.

## 1. Introduction

**Scheme 1:**
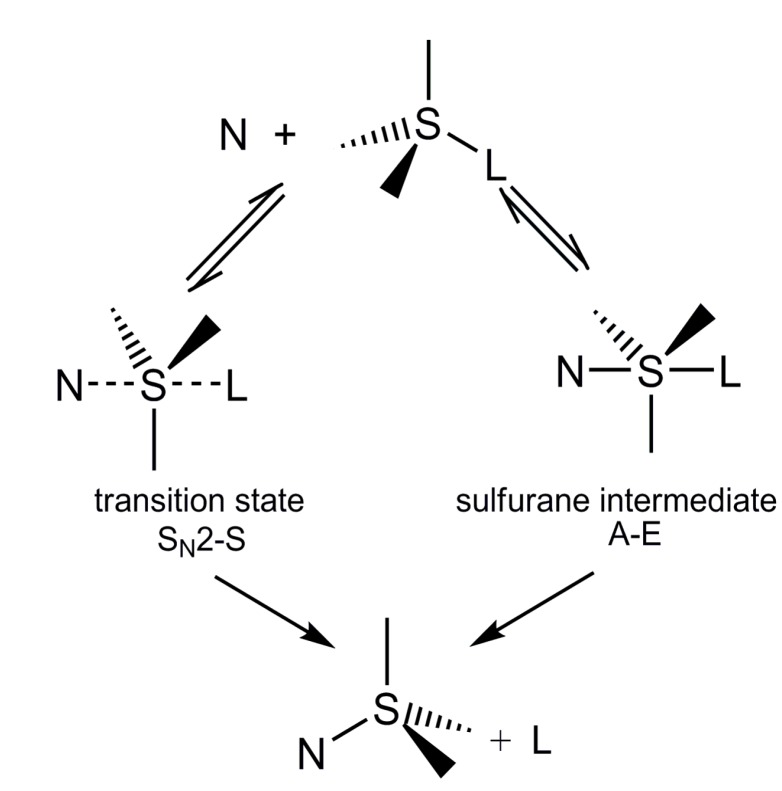
Possible mechanisms for nucleophilic substitution reactions at sulfur.

The mechanism and stereochemistry of nucleophilic substitution reactions at sulfur, S_N_-S, as well as at other heteroatoms (P, Si, Se, *etc.*), have been a subject of extensive studies of many research groups in recent decades [1]. Due to the fact that sulfur may form tetra- or pentacoordinate compounds (sulfuranes) [2,3], the most important question concerning the mechanism of S_N_-S reactions is whether these reactions occur synchronously according to an S_N_2-S mechanism or stepwise by an addition-elimination mechanism, A-E, involving sulfuranes as intermediates that are formed by addition of nucleophiles, N, to the reaction substrates (Scheme 1).

**Scheme 2:**
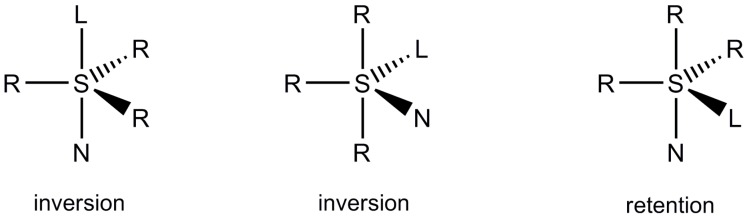
Relationship between the structure of transient sulfuranes and stereochemistry of A-E reactions at sulfur.

The second closely related problem is connected with the relationship between the structure of transiently formed sulfuranes and the stereochemical outcome of nucleophilic substitution reactions. It is now generally accepted that diaxial or diequatorial disposal of entering, N, and leaving, L, groups in a trigonal bipyramidal structure of transient sulfurane intermediates should lead to inversion of configuration at sulfur while the steric course of axial-equatorial substitution is predicted to be retention. (Scheme 2).

**Scheme 3:**
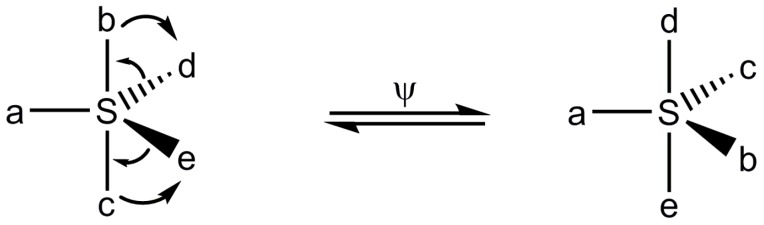
A single Berry pseudorotation process of a sulfurane structure.

However, the steric course of the S_N_-S reactions proceeding according to the A-E mechanism may also be affected by permutational isomerization of sulfuranes. This process, commonly called pseudorotation, consists in the internal ligand reorganization changing the relative positions of axial and equatorial ligands in a trigonal bipyramidal structure. A single pseudorotation process according to the Berry mechanism is shown below (Scheme 3). Since pseudorotation processes are of very low energy (the energy barriers are in the range from ca. 6 to 8 kcal/mol [4,5]), they may have important influence on the stereochemical outcome of nucleophilic substitution at sulfur which may vary from inversion to retention and racemization.

**Scheme 4:**
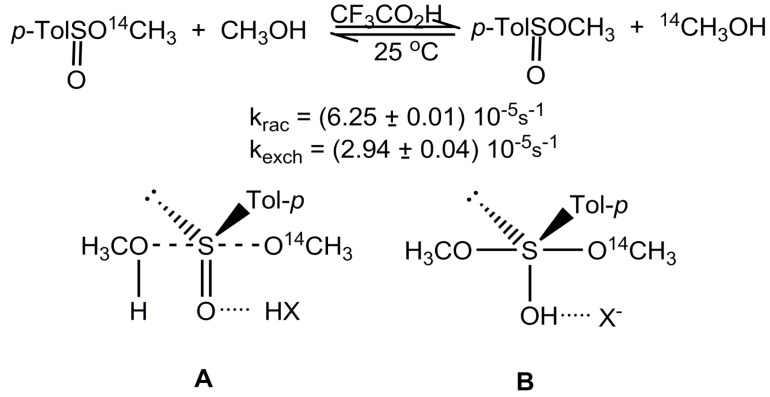
Acid-catalyzed methanolysis of optically active ^14^C-methyl *p*-toluenesulfinate and structures of transition state A and sulfurane B.

The majority of nucleophilic substitution reactions at the stereogenic sulfur atom occur with inversion of configuration. For example, this steric course has unequivocally been established in the reaction of optically active methyl *p*-toluenesulfinate containing ^14^C in the methoxy group with methanol catalyzed by trifluoroacetic acid (Scheme 4) [6]. The measurements of the rate of racemization of this sulfinate and the rate of isotopic methoxy-methoxy exchange revealed that it loses its optical rotation practically twice as fast as it loses the radiolabelled methoxy group. This finding provided a clear-cut evidence for a full inversion of configuration in the elementary process of the methoxy-methoxy exchange at the sulfinyl sulfur.

However, the observation of inversion in the above reaction, as well as in a great number of other S_N_ reactions at sulfur, does not allow to distinguish between the S_N_2-S and A-E mechanisms since both the transition state **A** and sulfurane intermediate **B** proposed for the methoxy-methoxy exchange at the chiral sulfinyl centre explain this steric course. The only conclusion, which can be drawn, is that the sulfurane **B**, if it is formed, should decompose before pseudorotation because all substituents around sulfur are properly placed in a trigonal bipyramidal structure from the viewpoint of apicophilicity.

**Scheme 5:**
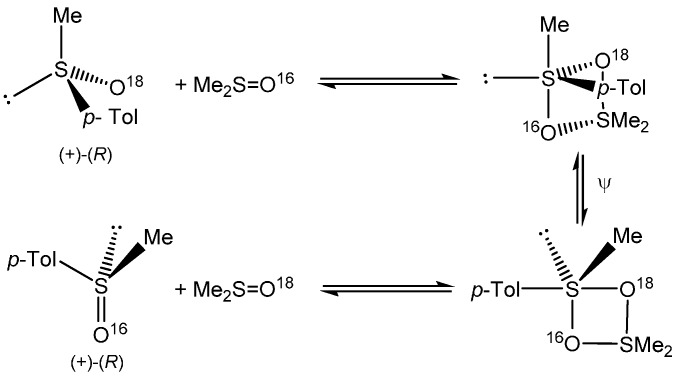
Steric course and mechanism of ^18^O/^16^O exchange in optically active methyl *p*-tolyl sulfoxide.

In contrast to stereoinvertive S_N_-S reactions, those occurring with retention at the sulfinyl sulfur give more convincing evidence for the operation of the A-E mechanism. In almost all such reactions reported so far retention at sulfur was convincingly explained by formation of a transient four-membered ring sulfurane intermediate with apical-equatorial arrangement of entering and leaving groups that undergoes pseudorotation and then decomposes to final product with retained configuration [7]. In accord with the microscopic reversibility rule, the pseudorotation of the primarily formed sulfurane intermediate is required to form a new sulfurane with the leaving group in apical position, however, without changing the preferable apical-equatorial disposal of a four-membered ring. The sulfur oxygen exchange between the ^18^O-labelled (+)-(*R*)-methyl *p*-tolyl sulfoxide and dimethyl sulfoxide proceeding without racemization, *i.e*., with retention, is the best example of the A-E mechanism discussed above (Scheme 5) [8].

In the course of our studies on static and dynamic stereochemistry of organic sulfur compounds, especially those with the sulfur atom as a sole centre of chirality, we became interested in the reactions of sulfinamides with alcohols catalyzed by acids (Equation (1)). We hoped that based on this reaction, completely unknown at the beginning of our work, a new and general synthetic approach to sulfinates can be devised. Moreover, since the starting sulfinamides were accessible in enantiomeric forms, this reaction should also provide a new access to optically active sulfinates. Apart from synthetic aspects, the acid catalyzed alcoholysis of sulfinamides has attracted our attention as a model reaction of nucleophilic substitution at the sulfinyl sulfur atom. Examination of its stereochemistry could gain further experimental insight into the complex nature of the S_N_-S reactions and give new evidence for the addition-elimination mechanism, A-E.

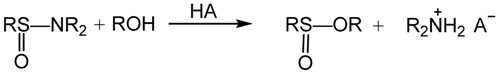
(1)

In this paper we wish to report the complete results of our detailed investigations of this reaction using a broad spectrum of optically active sulfinamides, alcohols and acidic catalysts and to rationalize the most interesting and unique discovery that its steric course varies from inversion to predominant retention and can be influenced by many factors. Preliminary results of our studies have been reported in two short communications [9,10].

## 2. Results and Discussion

### 2.1. Synthesis of Racemic Sulfinates

**Scheme 6:**
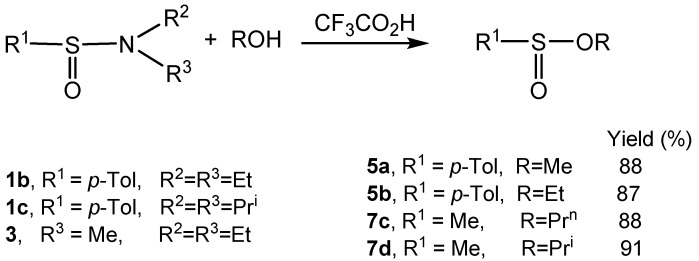
Synthesis of racemic sulfinates from sulfinamides.

At the outset of our studies, the synthetic value of the reaction under discussion was checked out using racemic sulfinamides as substrates. The latter were easily prepared by condensation of sulfinyl chlorides with amines. It was found that treatment of a series of racemic sulfinamides **1** and **3** with alcohols in the presence of trifluoroacetic acid afforded the corresponding sulfinates **5** and **7** in excellent yields (Scheme 6). In general, the reactions were carried out at 0 °C or at room temperature using an excess of alcohol and two molar equivalents of trifluoroacetic acid with respect to sulfinamide. Pure sulfinates were obtained by distillation or column chromatography.

Efficient and simple preparation of racemic sulfinates from sulfinamides paved the way for elaboration of a chiral version of this conversion in which optically active sulfinamides are used.

### 2.2. Synthesis of Optically Active Sulfinamides

**Figure 1:**
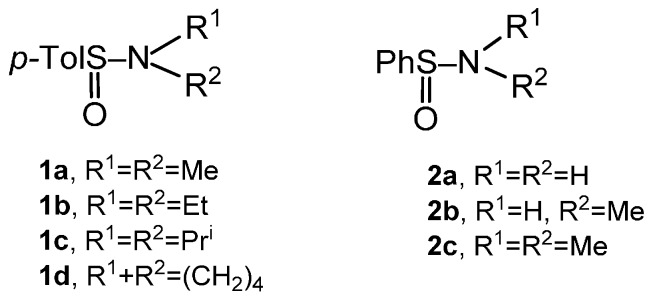
Optically active sulfinamides.

Having in mind the development of a general synthesis of optically active sulfinates and detailed examination of the stereochemistry of the sulfinamide→sulfinate conversion, a number of optically active sulfinamides were prepared. Their structures are shown below (Figure 1).

Optically active sulfinamides **1a**–**d** have been prepared essentially according to the method reported by Montanari *et al.* from the diastereoisomerically pure (–)-(*S*)-menthyl *p*-toluenesulfinate (**4**) and the appropriate dialkylaminomagnesium bromides [11]. This reaction was demonstrated to occur with inversion of configuration at sulfur (Equation 2).

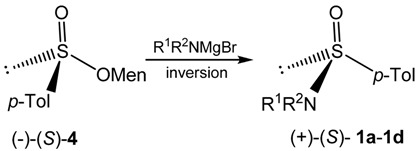
(2)

However, because in our hands this procedure gave results (see Table 1) different from those reported, it seems desirable to describe briefly our own observations. Firstly, we found that this reaction in THF-ethyl ether or ethyl ether solutions is very slow at −45 °C and occurs with a synthetically acceptable rate only at temperatures above 0 °C. Secondly, the reaction stereoselectivity was found to be dependent on the reaction temperature, structure of aminomagnesium bromides and sulfinamides formed. For example, when the reaction of sulfinate (–)-(*S*)-**4** with pyrrolidinemagnesium bromide was carried out at 0 °C the sulfinamide **1d** was isolated with [α]_D_ = +215 (88.8% op). The same reaction carried out at room temperature gave **1d** with much lower optical rotation, [α]_D_ = +135 (50.7% op), but in a comparable yield.

**Table 1:** Stereoselective synthesis of *p*-toluenesulfinamides **1** from (−)-(*S*)-menthyl *p*-toluenesulfinate (**4**).

Sulfinate 4	Reaction Conditions		Sulfinamide 1			
[α]_D_ (Me_2_CO)	Temp. (°C)	Time (h)	No	Yield (%) ^a^	[α]_D_ (EtOH)	(% op) ^b^
−210.0	25	15	**1a**	45	+5.5	3.5
−210.0	15	7	**1b**	41	+105.0	88
−210.0	15	20	**1c**	35	+104.3	54
−198.0	25	2	**1c**	65	+87.0	42
−202.0	0	15	**1d**	75	+215.0	81
−202.0	25	15	**1d**	70	+135.0	51

^a^ Yields after chromatography. ^b^ Optical purity values for **1a** and **1b** were calculated based on reference [11] and those for **1d** determined in this work.

The stereoselectivity of the reaction leading to the sulfinamide **1c**, although low, was found to be independent of the reaction temperature and **1c** turned out to be optically stable under the reaction conditions. However, our additional experiments showed that it undergoes decomposition in the presence of diisopropylmagnesium bromide. Therefore, the shorter reaction time resulted in a higher yield of **1c**. These observation taken altogether are most probably indicative of an addition-elimination mechanism responsible for the partial retention of configuration at sulfur during the replacement of the menthoxy group by the bulky diisopropylamino moiety. The reaction of the sulfinate (−)-(*S*)-**4** with dimethylaminomagnesium bromide carried out under our experimental conditions afforded the sulfinamide **1a** that was practically racemized. We believe that in this case the racemization of **1a** in the reaction medium is due to a competive symmetrical exchange of the dimethylamino group at the sulfinyl sulfur.

**Scheme 7:**
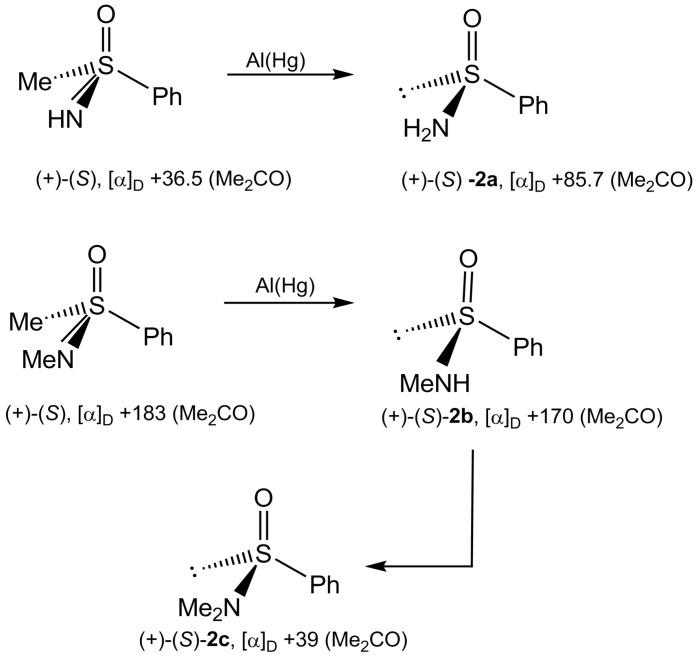
Synthesis of optically active sulfinamides **2**.

Optically pure (+)-(*S*)-benzenesulfinamide (**2a**) and (+)-(*S*)-*N*-methylbenzene sulfinamide (**2b**) have been obtained by reduction of the corresponding sulfoximides with aluminum amalgam according to and in a full agreement with the procedure reported by Johnson [12,13]. Optically active *N*,*N*-dimethyl derivative **2c** was prepared by methylation of the lithium salt of (+)-(*S*)-**2b** with methyl iodide at low temperature (Scheme 7), however, with a very low optical purity (15% op).

#### Interconversion of Sulfinamide Enantiomers

**Scheme 8:**
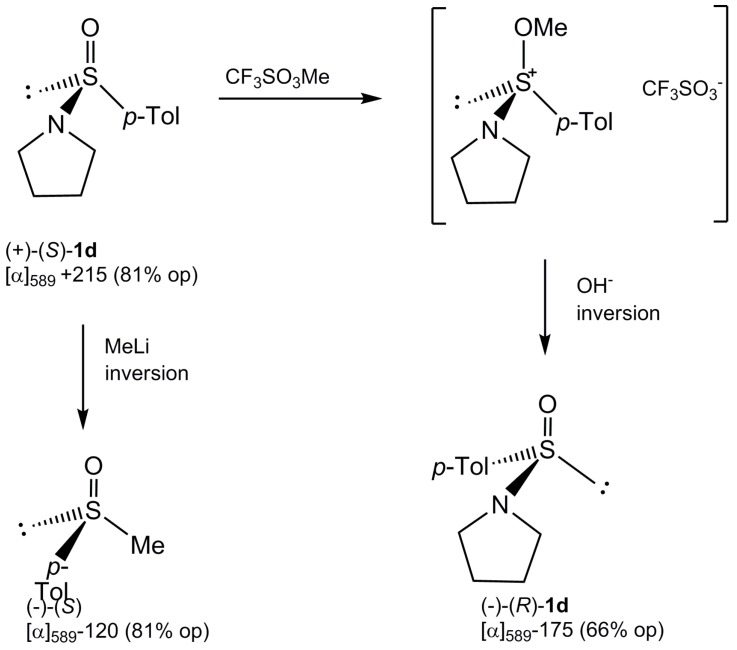
Interconversion of the *N*-*p*-tolylsulfinylpyrrolidine (**1d**) enantiomers.

The synthesis of optically active sulfinamides **1** from (–)-(*S*)-menthyl *p*-toluenesulfinate (**4**) described above afforded only the (+)-(*S*)-enantiomers of **1**. Therefore, in the course of our study and for the sake of its completeness it was desirable to find a way for the conversion of the sulfinamides (+)-(*S*)-**1** into their (–)-(*R*)-enantiomers. This could avoid the tedious preparation of the diastereoisomeric (+)-(*R*)-sulfinate **4**. Being stimulated by the original work by Johnson [14] on the interconversion of the sulfoxide enantiomers involving their *O*-methylation and subsequent alkaline hydrolysis, we decided to extend this approach to optically active sulfinamides **1**. In view of the fact that racemic *N*-*p*-tolylsulfinylpyrrolidine (**1d**) forms relatively stable *O*-alkoxysulfonium salts [15,16], the sulfinamide (+)-(*S*)-**1d**, [α]_D_ +215, was reacted with an excess of methyl triflate in nitromethane to give the corresponding methoxy-*N*-pyrrolidinyl-*p*-tolyl-sulfonium salt that was isolated and in a crude state hydrolysed under mild alkaline conditions affording (–)-(*R*)-sulfinamide **1d**, [α]_D_ −175. It is necessary to point out in this place that the absolute configuration and optical purity of the starting sulfinamide (+)-(*S*)-**1d** was established by its conversion accompanied by inversion of configuration at sulfur into the well known (–)-(*S*)-methyl *p*-tolyl sulfoxide with optical rotation value, [α]_D_ −120, which corresponds to 81% of optical purity. Hence, the optical purity of the sulfinamide (–)-(*R*)-**1d**, obtained is equal to 66%. Most probably the loss of stereoselectivity in the alkaline hydrolysis of sulfonium triflate is due to a competitive attack of the hydroxy anion at the methoxy carbon giving back the starting (+)-(*S*)-**1d**. The reactions discussed above are summarized in Scheme 8.

### 2.3. Stereoselective Synthesis of Optically Active Sulfinates and Stereochemistry of Their Formation

Having in hand the enantiomerically enriched sulfinamides **1** and **2** we could achieve the main goal of the present work *i.e*., the synthesis of optically active sulfinates and determination of the stereochemistry of their formation in the acid catalyzed alcoholysis of sulfinamides shown in Equation (3).

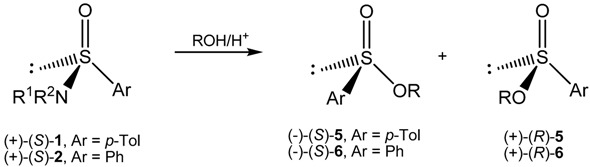
(3)

In general, the reaction of the optically active sulfinamides **1** and **2** with various alcohols was caried out at room temperature using a great excess of alcohols and two molar equivalents of acidic catalyst. The progress and termination of the alcoholysis was followed polarimetrically. The isolated, analytically pure sulfinates **5** and **6** were characterized by IR and NMR spectroscopy. Their optical purity and absolute configuration were estimated from the literature data or via their stereospecific conversion into optically active methyl *p*-tolyl sulfoxide [17,18,19].

In the first series of experiments (+)-(*S*)-*N*,*N*-diethyl *p*-toluenesulfinamide (**1b**) was reacted with primary, secondary and tertiary alcohols in the presence of strong acids to give the corresponding optically active sulfinates **5**, *p*-TolS(O)OR. In all the investigated cases the obtained **5** exhibited negative sign of optical rotation which points to their *S*-configuration at sulfur and formation with inversion of configuration. An inspection of the results of this set of experiments, which are summarized in Table 2, revealed that stereoselectivity of the conversion of (+)-(*S*)-**1b** into the sulfinates (−)-(*S*)-**5** is markedly dependent on the structure of alcohols. With primary alcohols, except benzyl alcohol, a full or almost full stereoselectivity was observed. The isopropanolysis reaction gave the corresponding sulfinates with the stereoselectivity from 58% to 84%. Interestingly, it was dependent to some extent on the nature of acidic catalyst. When *t*-butanol was used the reaction stereoselectivity was quite low. A similar gradual decrease in the reaction yields was found on going from primary to tertiary alcohols. Moreover, the reaction rates were also changed in the same direction. For example, the termination time of methanolysis estimated polarimetrically is 45 min, isopropanolysis requires 2.5 h to be completed, while the reaction with *t*-butanol is finished after 8 h.

**Table 2:** Stereoselective synthesis of optically active (+)-(*S*) *O*-alkyl *p*-toluenesulfinates (**5**) from sulfinamide (+)-(*S*)-**1b**.

Sulfinamide 1b			Acid	Sulfinate 5					Stereochemical Outcome	
No	[α]_D_ (Me_2_CO)	Op (%)		Yield (%)	No	R	[α]_D_ (EtOH)	Op (%)	Selectivity	Inversion (%)
**1b**	+107	88	CF_3_CO_2_H	94.0	**5a**	Me	−192.6	88	100	100.0
**1b**	+105	86	PhSO_3_H	76.5	**5b**	Et	−179.2	86	100	100.0
**1b**	+107	88	CF_3_CO_2_H	90.0	**5b**	Et	−137.5	66	75.5	87.7
**1b**	+105	86	PhSO_3_H	80.0	**5c**	Pr^n^	−161.2	84	98.2	91.1
**1b**	+106	87	PhSO_3_H	95.0	**5e**	CH_2_=CHCH_2_	−106.5	73	84.0	92.0
**1b**	+105	86	CF_3_CO_2_H	84.0	**5f**	HC=CCH_2_	−85.9	77	89.5	94.7
**1b**	+105	86	PhSO_3_H	77.0	**5g**	PhCH_2_	−22.6	88	30	65.0
**1b**	+96	78.5	CF_3_CO_2_H	87.0	**5d**	Pr^i^	−109.3	54	69.5	84.7
**1b**	+105	86	PhSO_3_H	53.0	**5d**	Pr^i^	−100.7	50	58	79.0
**1b**	+104.7	86	CF_3_CO_2_H	86.0	**5d**	Pr^i^	−107.9	54	62.7	81.5
**1b**	+105	86	HSbF_6_	61.0	**5d**	Pr^i^	−134.2	67	77	88.5
**1b**	+104.7	86	CF_3_CO_2_H	55.0	**5i**	Bu^i^	−29.85	23	27.4	63.7
**1b**	+104.7	86	CF_3_SO_3_+CF_3_CO_2_H	55.5	**5i**	Bu^i^	−10.6	8.3	9.7	54.8

These features of the alcoholysis of (+)-(*S*)-**1b** mentioned above are due to its partial racemization and decomposition under the acidic reaction conditions. For instance, when the reaction of (+)-(*S*)-**1b**, [α]_D_ +96 (78.5% op), with *t*-butanol was quenched at the half-conversion, it was recovered with much lower optical rotation equal to [α]_D_ +45 (39.8% op) whereas the sulfinate **5i** was isolated with almost the same optical rotation, [α]_D_ −32.5 (25% op), as that when the reaction was complete. Moreover, it was found that the sulfinamide (+)-(*S*)-**1b** in the presence of strong acids undergoes very fast racemization and decomposition in nonpolar solvents (CCl_4_, CHCl_3_) which occur slower in alcohols.

From the dynamic stereochemistry viewpoint, much more interesting results were obtained when (+)-(*S*)-*N*,*N*-diisopropyl *p*-toluenesulfinamide (**1c**) was used as a substrate in acid catalyzed alcoholysis reaction. In contrast to (+)-**1b**, (+)-**1c** was found to be optically and chemically stable under the acidic reaction conditions. In Table 3 the selected results of this series of experiments are summarized. Thus, with primary alcohols (MeOH, EtOH, ^n^PrOH) the laevorotatory (*S*)-sulfinates **5a**, **5b** and **5c** were formed with predominant inversion of configuration. However, the reaction of (+)-(*S*)-**1c** with isopropanol, its hexadeutero and hexafluoro analogues, cyclohexanol and cyclopentanol afforded unexpectedly the corresponding dextroratory sulfinates **5d**, **5d**', **5d**'', **5j** and **5k** with predominant retention of configuration. The percentage of retention was especially high with cyclohexanol (74.5%). These findings show clearly that steric factors in the attacking alcohol and departing dialkylamino group exert important influence on the stereoselectivity and, first of all, on steric course of the investigated reaction. Therefore, combination of a sterically hindered alcohol as a nucleophile and a bulky leaving diisopropylamino group is mainly responsible for the reversal of stereochemistry from inversion to retention.

**Table 3:** Stereoselective synthesis of optically active *O*-alkyl *p*-toluenesulfinates (**5**), *p*-TolS(O)OR, from sulfinamide (+)-(*S*)-**1c** using trifluoroacetic acid as catalyst.

Sulfinamide 1c		Sulfinate 5				Inversion (%) or Retention (%)
[α]_D_ (Me_2_CO)	Op (%)	No	R	[α]_D_ (EtOH)	Op. (%)	
+94.4	45.3	**5a**	Me	−35.0	16.0	68.75, Inv
+94.4	45.3	**5b**	Et	−35.0	3.4	53.75, Inv
+94.4	45.3	**5c**	Pr^n^	−13.9	7.3	58.0, Inv
+94.4	45.3	**5d**	Pr^i^	+15.8	7.9	58.7, Ret
+86.9	42.3	**5d'**	(CD_3_)_2_CH	+10.1	4.6	55.5, Ret
+86.9	42.3	**5h**	(CF_3_)_2_CH	+3.4	1.7	52.0, Ret
+95.0	45.3	**5j**	Hex^c^	+41.0	22.4	74.5, Ret
+95.0	45.3	**5k**	Pen^c^	+3.3	1.8	52.0, Ret
+95.0	45.3	**5l**	Et_2_CH	−4.4	2.3	52.5, Inv
+95.0	45.3	**5m**	Bu^i^	−2.7	1.4	51.5, Inv

In an extention of the present work the reaction of optically active benzenesulfinamides (+)-(*S*) **2a**–**c** with methanol and ethanol catalyzed by trifluoroacetic acid was investigated. As the results collected in Table 4 show, the sulfinates (–)-**6** were formed with inversion of configuration and variable degree of stereoselectivity. A full inversion of configuration was observed with (+)-(*S*)-*N*,*N*-dimethyl benzenesulfinamide (**2c**).

**Table 4:** Stereoselective synthesis of optically active *O*-alkyl benzenesulfinates (**6**), PhS(O)OR, from benzenesulfinamides (+)-(*S*)-**2** using trifluoroacetic acid as catalyst.

Sulfinamide 2			Sulfinate 6				Inversion (%)
No	[α]_D_ (Me_2_CO)	Op (%)	No	R	[α]_D_ (EtOH)	Op. (%)	
**2a**	+85.7	100	**6a**	Me	−64.0z	24.0	62.0
**2a**	+85.7	100	**6b**	Et	−23.0	10.8	55.4
**2b**	+170.0	100	**6a**	Me	−204.2	76.5	87.2
**2b**	+170.0	100	**6b**	Et	−167.0	78.0	89.0
**2c**	+39.0	15.6	**6a**	Me	−45.5	15.6	100.0
**2c**	+39.0	15.6	**6b**	Et	−33.4	15.6	100.0

**Scheme 9:**
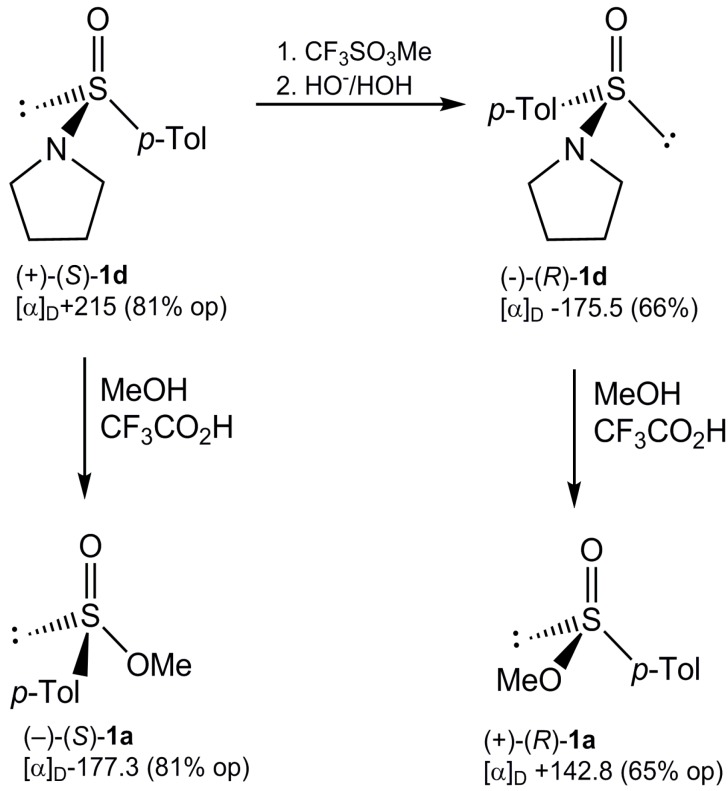
Synthesis of enantiomeric O-methyl *p*-toluenesulfinates (**1a**) from *N*-*p*-tolylsulfinylpyrrolidines (**1d**).

Having in hand both enantiomerically enriched *N*-*p*-toluenesulfinylpyrrolidines (+)-(*S*)-**1d** and (–)-(*R*)-**1d** prepared as shown in Scheme 8, we were also able to determine the steric course of their methanolysis. It turned out that in this case the corresponding enantiomeric sulfinates **5a** were formed in a stereospecific way with inversion of configuration. It is worthy to point out that the synthesis of both enantiomeric methyl sulfinates **5a** (Scheme 9) demonstrates how they can be prepared starting from only one form of the diastereoisomeric menthyl *p*-toluenesulfinate (–)-(*S*)-**4**.

The stereochemistry of the acid-catalyzed alcoholysis of sulfinamides may also be affected by addition of silver perchlorate and other inorganic salts. In a preliminary experiment we found that isopropanolysis of (+)-(*S*)-*N*,*N*-diethyl *p*-toluenesulfinamide (**1b**) catalyzed by trifluoroacetic acid and carried out in the presence of silver perchlorate occurred with a higher stereoselectivity (92% inversion) than that observed in its absence (84.7% inversion). Being stimulated by this observation, a detailed study was undertaken on the effect of added inorganic salts on the steric course of the acid catalyzed alcoholysis of the sulfinamide (+)-(*S*)-**1c** (Equation (4)). The results are outlined in Table 5.

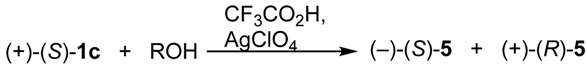
(4)

**Table 5:** The effect of silver perchlorate on steric course of the acid catalyzed alcoholysis of sulfinamide (+)-(*S*)-**1c**.

Sulfinate 5	Inversion/Retention Ratio with AgClO_4_	Inversion/Retention Ratio without AgClO_4_
**5a**, Me	100/0	68.7/31.3
**5b**, Et	91/9	53.7/46.3
**5c**, Pr^n^	100/0	58.0/42.0
**5d**, Pr^i^	82/18	41.3/58.7
**5j**, Hex^c^	65.5/34.5	25.5/74.5

As it is seen, silver perchlorate favours the formation of the sulfinates **5** with inversion of configuration. The most impressive change was observed with isopropanol and cyclohexanol which reacted with (+)-(*S*)-**1c** in the absence of this inorganic salt with prevailing retention of configuration.

The effect of silver perchlorate discussed above prompted us to investigate the stereochemistry of the acid catalyzed reaction of the sulfinamide (+)-(*S*)-**1c** with isopropanol in the presence of other inorganic salts (Equation (5)). The results of this set of experiments are collected below.

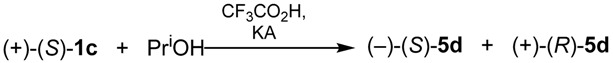
(5)

An inspection of the results collected in Table 5 and Table 6 demonstrates that the added inorganic salts are able to radically change the overall stereochemistry of the investigated reaction and both the cation (K) and anion (A) play an important role in this regard. In other words, a new tool was found which allows us to control and design the reaction stereochemistry.

**Table 6:** The effect of inorganic salts (KA) on the steric course of the acid catalyzed isopropanolysis of sulfinamide (+)-(*S*)-**1c**.

KA	Prevailing Stereochemistry	KA	Prevailing Stereochemistry
CoCl_2_	55% Retention	Co(NO_3_)_3_	73.0% Inversion
NiC_2_O_4_	71% Retention	Ni(NO_3_)_2_	66.0% Inversion
Ag_2_CO_3_	65% Retention	AgClO_4_	82.0% Inversion
Ag_2_Cr_2_O_7_	67% Retention	AgNO_3_	53.0% Inversion
Ag_2_SO_4_	63% Retention	Ce(NO_3_)_3_	71.0% Inversion
HgBr_2_	69% Retention	CrCl_3_	50.5% Inversion
Cd(OAc)_2_	68% Retention		

In contrast to significant influence of inorganic salts, the reaction stereochemistry was found to be slightly solvent dependent (see Equation (6) and Table 7). Although only four solvents were tested, it seems that polar solvents may favour retention of configuration.

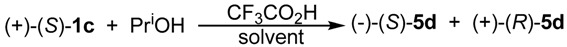
(6)

**Table 7:** The effect of solvents on steric course of the acid catalyzed isopropanolysis of sulfinamide (+)-(*S*)-**1c**.

Solvent	Inv/Ret Ratio
CHCl_3_	55/45
C_6_H_6_	56/46
C_6_H_14_^n^	58/42
CH_3_CN	49/51

### 2.4. Reaction Kinetics

The observation of a unique stereochemistry of the acid catalyzed alcoholysis of optically active sulfinamides **5**, which may vary from inversion to predominant retention of configuration at the stereogenic sulfur atom, and its sensitivity to internal (the structure of both reactants) and external (the presence of inorganic salts) factors prompted us to determine the reaction kinetics as integral part of the present study aimed at elucidation of the reaction mechanism.

Two model reactions were chosen for kinetic investigations *i.e*., isopropanolysis of (+)-(*S*)-*N*,*N*-diethyl *p*-toluenesulfinamide (**1b**) and *N*,*N*-diisopropyl *p*-toluenesulfinamide (**1c**) with the presence of trifluoroacetic acid. The latter was used in two molar excess (0.11752 mol/L) in respect to sulfinamide (0.05876 mol/L) and both reactions were carried out in isopropanol as a solvent used in 200 molar excess. The progress of the isopropanolysis reaction was followed polarimetrically. The calculated pseudo-first order rate constants at various temperatures (298–318 K) are listed in Table 8. For comparison purposes, the pseudo-first order rate constant of the reaction of (+)-(*S*)-*N*,*N*-dimethyl *p*-toluenesulfinamide (**1a**) at 310 K was determined. Based on the variable temperature measurements the energy and entropy of activation (at 25 °C) have been calculated and are shown in Table 9:

**Table 8:** Kinetic data on alcoholysis of arenesulfinamides, ArS(O)NR^1^R^2^, catalyzed by trifluoroacetic acid.

Run	Sulfinamide		Alcohol	Temp. (K)	k_1_(10^−4^s^−1^)
**1**	**1a**,	*p*-TolS(O)NMe_2_	Pr^i^OH	310.0	16.0 ± 0.35
**2**	**1b**,	*p*-TolS(O)NEt_2_	Pr^i^OH	298.0	2.32 ± 0.35
**3**	**1b**,			307.1	3.75 ± 0.09
**4**	**1b**,			310.1	5.22 ± 0.10
**5**	**1b**,			318.0	8.64 ± 0.15
**6**	**1b**,			328.0	19.5 ± 0.30
**7**	**1b**,	*p*-TolS(O)NEt_2_	MeOH	298.0	33.0 ± 1.2
**8**	**1b**,	*p*-TolS(O)NEt_2_	CH_3_OD	298.0	47.9 ± 1.5
**9**	**1c**,	*p*-TolS(O)NPr_2_^i^	Pr^i^OH	303.7	0.521 ± 0.015
**10**	**1c**,			310.0	0.935 ± 0.04
**11**	**1c**,			316.7	1.57 ± 0.04
**12**	**2**,	*p*-TolS(O)NMe_2_	Pr^i^OH	310.0	5.60 ± 0.10

**Table 9:** Activation energy and entropy for isopropanolysis of sulfinamide **1b** and **1c** catalyzed by trifluoroacetic acid.

Reaction	Ea (kJ mol^−1^/kJ mol^−1^)	ΔS^≠^ (J mol^−1^ k^−1^/e.u.)
**1b**	58.3/14	−145.5/−34.8
**1c**	68.1/16.3	−110.4/−26.4

The values of energy and entropy of activation are characteristic for a typical bimolecular substitution reaction. The gradual decrease in the reaction rate constants measured at 310 K on going from isopropanolysis of **1a** (k = 16 ± 0.35) to **1b** (k = 5.22 ± 0.1) and to **1c** (k = 0.935 ± 0.04) indicates that an increase of steric bulk at the amido-nitrogen atom is responsible for these changes. Similary, the rate constant (at 298 K) of isopropanolysis of **1b** (k = 2.32 ± 0.08) is much smaller than that for methanolysis of **1b** (k = 33 ± 1.2) determined at the same temperature. In this case, the difference in rate constants is due to introduction of a steric bulk to reacting alcohol as a nucleophilic reagent. Such a relationship between reaction rate constatnts and steric hindrance is typical for bimolecular nucleophilic substitution reactions.

In addition to calculation of the rate constant for the reaction of (+)-(*S*)-**1b** with methanol, that plays here dual role of a nucleophile and a solvent, the corresponding rate constant in deuterated methanol, CH_3_OD, was estimated. This allowed us to calculate the kinetic isotopic effect of solvent equal to 1.45.

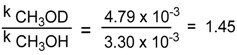
(7)

This value of kinetic isotopic effect of solvent points also to bimolecular reaction mechanism. Moreover, it indicates that protonation is the first and fast reaction step and does not determine the reaction rate. Interestingly, a very similar value of the kinetic isotopic effect of solvent was reported by Tillet who investigated hydrolysis of N-aryl arenesulfinamides under acidic conditions [20]. Moreover, the recent investigation of the hydrolysis rates of N-alkyl and N-aryl methanesulfinamides led the authors [21] to the conclusion that if nitrogen protonation does occur, it is not the rate-limiting step.

In order to rationalize our most interesting observation of predominant retention of configuration at sulfur in the reaction of the sulfinamide (+)-(*S*)-**1c** with isopropanol catalyzed by trifluoroacetic acid we took into consideration a possible two-step mechanism for this reaction involving the formation of a mixed anhydride as intermediate product (Equation (8)). Assuming that its formation and subsequent isopropanolysis could occur with inversion of configuration, the sulfinate (+)-(*R*)-**5d** should be formed with retention of configuration.

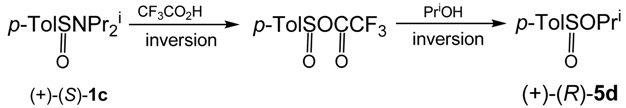
(8)

To support or rule out this mechanistic possibility, the rate constant and steric course of the reaction were determined at various concentrations of the added sodium trifluoroacetate (Equation (9)). It was anticipated that the presence of trifluoroacetate anion should facilitate the formation of mixed anhydride and increase the percentage of retention.

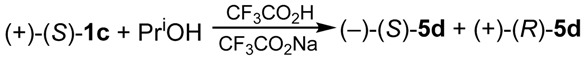
(9)

As it is seen in Table 10, the results of the above kinetic measurements are not consistent with the hypothesis of a two-step mechanism involving mixed anhydride and double inversion. Therefore, the diverse stereochemistry of the reaction under discussion is most probably due to a competition between the inversion and retention processes.

**Table 10:** The effect of concentration of sodium trifluoroacetate on the rate constants and stereochemistry of the reaction 9.

Concentration of CF_3_CO_2_Na	Rate Contant	Prevailing Stereochemistry
0 mol	k = (2.73 ± 0.26) 10^−4^ s^−1^	58% Ret
16 mol	k = (3.29 ± 0.06) 10^−4^ s^−1^	58% Inv
32 mol	k = (3.56 ± 0.18) 10^−4^ s^−1^	59% Inv

### 2.5. Discussion

Before discussing the mechanism of the acid catalyzed alcoholysis of sulfinamides it is necessary to emphasize that the stereochemistry of this reaction studied with optically active sulfinamides shows unique features. Namely, it was found that optically active sulfinates are formed with a full or predominant inversion or predominant retention of configuration at stereogenic sulfur. The predominant retention of configuration was observed with bulky dialkylamido groups in sulfinamides and with sterically demanding alkyl substituents in alcohols. Moreover, the stereochemical outcome of the alcoholysis reaction may be changed from inversion to retention and *vice versa* by adding inorganic salts to the reaction medium. To a lesser extent the steric course is influenced by the nature of acid catalysts and solvents.

On the other hand, in contrast to the unusual stereochemistry, kinetic measurements revealed that the acid catalyzed alcoholysis of sulfinamides is a typical bimolecular substitution reaction at sulfur and protonation is a fast and not rate-detemining step. Although the sulfinamide molecule may be protonated on the nitrogen and oxygen atoms, it is evident that the nitrogen atom is protonated because in this way a leaving dialkylammonium group is created. Our comparative studies on the spectral properties of neutral and protonated sulfinamides led us to the same conclusion [22].

**Figure 2:**
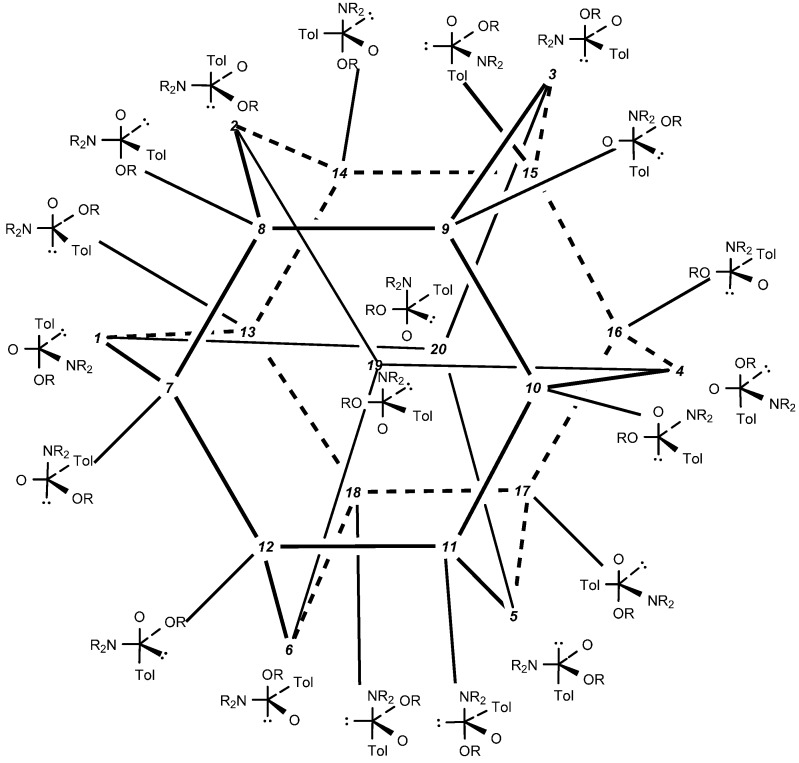
Hexaasterane graph showing all possible sulfuranes ***1*** to ***20*** internconnected by pseudorotations (for the sake of clarity hydrogens on the nitrogen and oxygen atoms are omitted).

All the stereochemical observations on our reaction summarized above may be best rationalized in terms of the addition-elimination mechanism, A-E, involving sulfurane intermediates that are able to undergo pseudorotation. Theoretically, addition of an alcohol to the protonated sulfinamide may results in the formation of twenty chiral sulfuranes interconnected by thirty pseudorotations. Four sulfuranes are formed by nucleophilic attack of an alcohol on four different walls of the protonated (*S*)-sulfinamide tetrahedron. Another six sulfurane structures result from the attact of an alcohol on six edges of the tetrahedron. The remaining ten sulfuranes are enantiomeric structures which may be derived from the (*R*)-sulfinamide. All these twenty sulfuranes are in equilibrium due to a very low energy for pseudorotation. They are displayed in the form of a hexaasterane graph, originally proposed by Mislow [23] for pentacoordinate phosphoranes, which was applied by us to discuss the stereochemical outcome (retention or inversion) of the alcoholysis of sulfinamides (Figure 2).

**Scheme 10:**
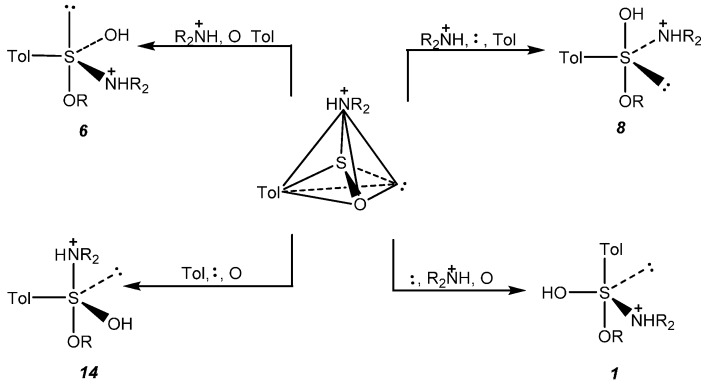
Sulfuranes formed by nucleophilic attack (apical entry) on four walls of the protonated (*S*)-sulfinamide **1** tetrahedron.

As it is now generally accepted that in nucleophilic substitution reactions apical entry and apical departure are preferred over the equatorial counterparts [24], only four sulfuranes (***1***, ***6***, ***8***, and ***14***) resulting from the attack on four walls remain as candidates for the initial products of addition of an alcohol to (*S*)-sulfinamide (Scheme 10).

**Scheme 11:**
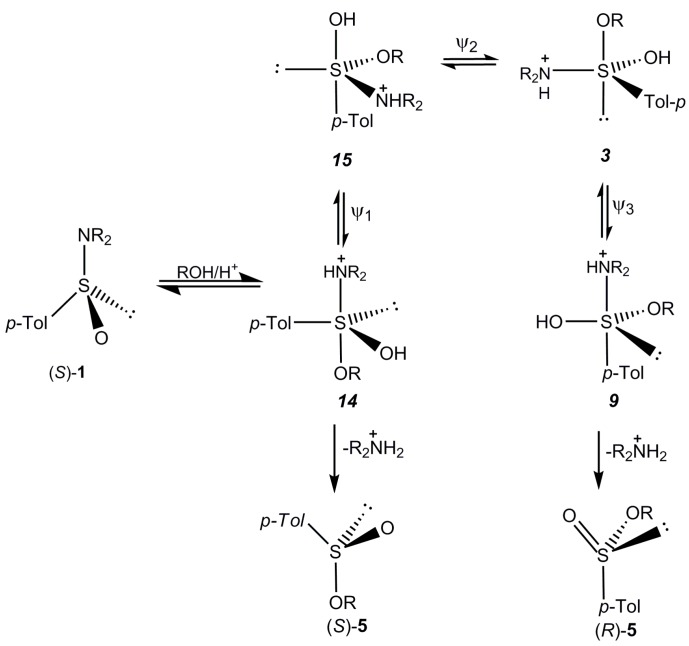
Two competing inversion and retention pathways in the acid catalyzed alcoholysis of sulfinamides **1**.

In further considerations we assumed that, after addition, the negatively charged sulfinyl oxygen atom is protonated. Among these four structures the highest probability of formation should have the sulfurane structure ***14*** because arrangement of substituents in a trigonal bipyramid is optimal from the viewpoint of apicophilicity of ligands. Due to the diapical disposal of the entering alkoxy group and leaving protonated dialkylamido group its direct decomposition should afford sulfinic acid ester **5** as a substitution product with inverted configuration at sulfur. In fact, this steric course has been observed for the reaction of alcohols with (+)-(*S*)-*N*,*N*-diethyl *p*-toluenesulfinamide (**1b**) and with (+)-(*S*)-benzenesulfinamide (**2a**) (+)-(*S*)-*N*-methyl benzenesulfinamide (**2b**) and (+)-(*S*)-*N*,*N*-dimethyl benzenesulfinamide (**2c**). Interestingly, the acid catalyzed methanolysis of both enantiomers of *N*-*p*-tolyslsulfinylpyrrolidine (**1d**) was occurring with a full inversion of configuration. However, as it was described earlier, the reactions (+)-(*S*)-*N*,*N*-diisopropyl *p*-toluenesulfinamide (**1c**) with secondary alcohols gave the corresponding sulfinates **5** with predominant retention of configuration. In this case, in the initially formed sulfurane ***14*** apical positions are occupied by two bulky groups, namely, the protonated diisopropylamino group having a tetrahedral structure and bulky alkoxy group. It is reasonable to expect that steric repulsive interactions between the latter groups and equatorial substituents (a-e angle ~ 90°) force the sulfurane ***14*** to pseudorotate to a new trigonal bipyramidal structure where a number of unfavourable interactions will be diminished. Thus, the pseudorotation of ***14*** using the lone electron pair as a pivot leads to the sulfurane **15** where two bulky dialkylammonium group and alkoxy substituent are placed in equatorial positions (e-e angle ~110°), and the steric interactions of apical and equatorial substituents are smaller. The next pseudorotation of ***15*** with the dialkylammonium group as a pivot results in the formation of the sulfurane ***3***. In this structure the unfavourable apical position of the lone electron pair is compensated by the right apical placement of a strongly apicophilic alkoxy group. To complete the retention pathway it is necessary to put the departing dialkylammonium moiety into apical position via pseudorotation of ***3***. The resulting sulfurane ***9*** decomposes to the sulfinate **5** with retention of configuration. The most probable and shortest pathways for inversion and retention are shown in Scheme 11.

The relative stability and concentration of the sulfuranes ***14*** and ***9*** will determine the ratio between the sulfinates (−)-(*S*)-**5** and (+)-(*R*)-**5** formed.

**Scheme 12:**
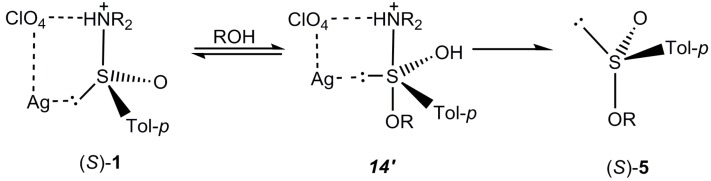
Preferred steric course of the acid catalysed alcoholysis of sulfinamides in the presence of silver perchlorate.

The important effect of the added inorganic salts on the steric course of the acid catalyzed alcoholysis of sulfinamides is, at present, very difficult to rationalize and requires further studies. Although it is evident that sulfinamides and their protonated species may form complexes with inorganic salts, their structures are unknown and sometimes hard to predict. As in the case of protonation of sulfinamides, the salt cations may be coordinated to three different sulfinamide sites, to sulfur, oxygen or nitrogen (Scheme 12).

Most probably silver perchlorate, that has been found to strongly prefer the inversion pathway, is coordinated to the protonated sulfinamide **1** in such a way that silver cation, as a soft metal ion, is bound to sulfur via its electron pair, while perchlorate anion, still being under control of silver cation, forms hydrogen bond with the ammonium nitrogen atom. Addition of alcohol to this complex affords the sulfurane ***14***' with the same mode of silver perchlorate coordination. In this way, the energy for pseudorotation of ***14***' is increased as compared with that of uncomplexed ***14*** and the direct apical departure of the dialkylammonium moiety is facilitated affording the sulfinates **5** with inverted configuration. However, the effect of other salts on the stereochemical outcome of our reaction is still obscure and should be investigated. Finally, it is necessary to point out that formation of sulfurane intermediates was postulated not only in the nucleophilic substitution reactions at the stereogenic sulfur atom [25,26] but also in many diverse chemical reactions [27,28,29,30,31,32] and enzymatic biotransformations [33] of organic sulfur compounds.

## 3. Experimental Section

### 3.1. General

Melting and boiling points are uncorrected. THF was distilled over K/benzophenone and benzene was distilled over Na wire, both immediately before use. Chloroform was distilled over P_2_O_5_ and stored over anhydrous Na_2_CO_3_. Thin layer chromatography (TLC) was conducted on Silica Gel 60 F_254_ TLC purchased from Merck (Darmstadt, Germany). Column chromatography was performed with Merck Silica gel (200–300 mesh). NMR spectra were recorded at 20 °C with Jeol C 60 HL, Tesla BS-487 C and Bruker HX 90 (Karlsruhe, Germany). ^1^H-NMR chemical shifts are reported relative to TMS as internal starndard. IR spectra were recorded with Specord 71 IR Carl Zeiss and Perkin-Elmer spectrophotometers (Jena, Germany). Optical rotations were measured at 20 °C using a Perkin-Elmer 141 photopolarimeter. All reactions under anhydrous conditions were carried out under a dry argon atmosphere. Elemental analyses were done in the Microanalytical Laboratory of the institute. The correct microanalysis data (H, ±0.3%, C, ±0.4%, S, ±0.4%) were obtained for all new compounds prepared in this work.

### 3.2. Synthesis of Optically Active p-toluenesulfinamides **1**

#### General Procedure

To a stirred solution of *n*-propylmagnesium bromide (0.03 mol) in ethyl ether (50 mL) a solution of the proper amine (0.03 mol) in ethyl ether (20 mL) was added at room temperature. After 20 min, a solution of (–)-menthyl *p-*toluenesulfinate (**4**) (0.01 mol) in ethyl eter (20 mL) was added at given temperature. The reaction mixture was stirred for the appropriate time (see below). Then, the reaction solution was washed with a saturated aqueous solution of NH_4_Cl (2 × 40 mL), 3% aqueous solution of HCl (1 × 20 mL) and 5% aqueous solution of Na_2_CO_3_ (2 × 20 mL). The organic layer was dried with MgSO_4_ and the solvent evaporated. The reaction product **1** was purified by column chromatography. Analytically pure products **1** were characterized by ^1^H-NMR and IR spectroscopy.

*(+)-(S)-N,N-Dimethyl-p-toluenesulfinamide* (**1a**)*.* The reaction of (−)-**4**, [α]_D_ = −210, (acetone) with dimethylaminomagnesium bromide was carried out at room temperature (25 °C) for 15 h. The crude product isolated as described above was purified by column chromatography (ethyl ether/*n*-hexane, 1:2) to afford the pure sulfinamide **1a** in 45% yield; [α]_D_ = +5.5 (c, 1.65, EtOH) (3.5% op); ^1^H-NMR (60 MHz, CDCl_3_) δ 7.45 and 7.11 (AB-system, 4H, H-Ar, *J*_AB_ = 8 Hz); 2.85 (s, 6H, (CH_3_)_2_N); 2.35 (s, 3H, CH_3_-Ar); IR (film, cm ^−1^) ν 1070, 1085 (S=O).

*(+)-(S)-N,N-Diethyl-p-toluenesulfinamide* (**1b**)*.* The reaction of (−)-**4**, [α]_D_ = −210 (acetone) with *N*,*N*-diethylaminomagnesium bromide was carried out at 15 °C for 7 h. The crude product was purified by column chromatography (silica gel, ethyl eter/*n*-hexane, 1:2) to give (+)-(*S*)-**1b**, as a colourless liquid in 41% yield; [α]_D_ = +105 (c, 1.48, EtOH) (88% op). ^1^H-NMR (60 MHz, CDCl_3_) δ 7.45 and 7.20 (AB-system, 4H, H-Ar, J_AB_=8.1 Hz); 3.00 (q, 4H, (CH_3_CH_2_)_2_N, *J* = 7.0 Hz); 2.35 (s, 3H, CH_3_-Ar); 1.10 (t, 6H, (CH_3_CH_2_)N, *J =* 7Hz); IR (film, cm^−1^), ν 1060, 1080 (S=O).

*(+)-(S)-N,N-Diisopropyl-p-toluenesulfinamide* (**1c**)*.* The raction of (−)-**4**, [α]_D_ = −210 (acetone), with diisopropylmagnesium chloride carried out at 15 °C for 20 h produced sulfinamide **1c** which after typical work up was purified by column chromatography (ethyl ether/*n*-hexane, 1:4). The pure sulfinamide **1c** as a liquid was obtained in 35% yield; [α]_D_ = + 104.3 (c, 1.12; EtOH) (50% op); ^1^H-NMR (60MHz, CDCl_3_) δ 7.35 and 7.15 (AB-system, 4H, H-Ar, *J*_AB_ = 8.0 Hz); 3.43 (sp, 2H, (CH_3_)_2_CHN, *J =* 8Hz); 2.35 (s, 3H, CH_3_-Ar); 1.35 (d, 6H, (CH_3_)_2_CHN, *J =* 6.5 Hz); 1.05 (d, 6H, (CH_3_)_2_CHN, *J =* 6.5 Hz); IR (toluene, cm^−1^) ν 1115 (S=O).

*(+)-(S)-N-p-Tolylsulfinylpyrrolidine* (**1d**)*.* Treatment of (−)-**4**, [α]_D_ = −202 (acetoe) with pyrrolidinemagnesium bromide at 0 °C for 15 h gave the desired sulfinamide **1d** in 74% yield; [α]_D_ = +215 (c, 1.5; EtOH) (80.8% op); mp. 43–49 °C; ^1^H-NMR (60 MHz, CD_3_NO_2_) δ 7.53. and 7.10 (AB-system, 4H, H-Ar, *J*_AB_ = 8 Hz); 3.41–2.57 (m, 4H, (CH_2_CH_2_)_2_N); 2.27 (s, 3H, CH_3_-Ar); 1.85–1.55 (m, 4H, (CH_2_CH_2_)_2_N);

*(−)-(S)-Methyl p-tolyl sulfoxide from* (+)-(*S*)-**1d***.* To a solution of methyllithium (0.015 mol) in ethyl ether (20 mL) a solution of (+)-(*S*)-**1d**, [α]_D_ = 215, (0.005 mol) in ethyl ether (20 mL) was added at room temperature. After 2 h stirring at room temperature, the reaction mixture was quenched with water. The separated ether layer was washed with water (2 × 10 mL) and the combined water layers were extracted with *n*-hexane (3 × 25 mL) and then with chloroform (3 × 25 mL). The chloroform extracts were dried over MgSO_4_. After evaporation of the solvent, the title sulfoxide was obtained by TLC (ethyl ether/*n*-hexane, 5:1) in 70% yield; [α]_D_ = −119.6 (c, 1.1, ethanol) (80.8% op), mp 71–73 °C, ^1^H-NMR (60 MHz, CDCl_3_) δ 7.50 and 7.30 (AB-system, 4H, H-Ar, *J*_AB_ = 8 Hz), 2.60 (s, 3H, CH_3_-SO), 2.35 (s, 3H, CH_3_-Ar); IR (KBr) ν 1060, 1080 (S=O).

*(+)-(S)-Benzenesulfinamide* (**2a**). According to the procedure described by Johnson, the reduction of (+)-(*S*)-S-methyl-S-phenyl-sulfoxyimine [α]_D_ = +36.5 (c, 2.0, acetone) with aluminum amalgam was carried out in a water-THF solution (1:9) for 2 h. After standard work up, the crude reaction product was purified by chromatography (silica gel 200–300 mesh, acetone/*n*-heptane, 2:1) to give (+)-(*S*)-**2a** as a white solid, mp. 95–101 °C, [α]_D_ = +85.7 (c, 1.82; acetone) [lit. [10], [α]_D_ = 82.9; mp. 102–103 °C after crystallization from ethyl ether]; ^1^H-NMR (60MHz, CDCl_3_) δ 7.90–7.35 (m, 5H, H-Ar); 3.45 (s, 2H, H_2_N).

*(+)-(S)-N-Methyl benzenesulfinamide* (**2b**). Reduction of (+)-(*S*)-N,S-dimethyl-S-phenyl-sulfoximine, [α]_D_ = +183 (c, 1.5 acetone), with aluminum amalgam in a H_2_O-THF (1:9) carried out as described above gave the desired product **2b** which, after stardard work up, was purified by chromatography (silica gel 200–300 mesh), acetone/*n*-heptane, 3:2) to give the pure (+)-(*S*)-**2b** as a solid, mp. 49–52 °C; [α]_D_ = 170 (c, 1.21; acetone); ^1^H-NMR (60 MHz, CD_3_OD) δ 7.80–7.45 (m, 5H, H-Ar); 4.80 (s, 1H, NH); 2.40 (s, 3H, CH_3_-Ar).

*(+)-(S)-N,N-Dimethyl benzenesulfinamide* (**2c**)*.* To a solution of (+)-(*S*)-**2b** (0.01 mol) in THF a solution of *n*-butyllithium (0.01 mol) in *n*-hexane was added at −70 °C. The reaction mixture was stirred at this temperature for 1 h. Then, methyl iodide (0.01 mol) was added and stirring was continued for additional 1 h, warming slowly the reaction solution to room temperature (20 °C). The reaction solution was washed with water (25 mL) and the organic layer dried over dry MgSO_4_. Removal of the solvent gave the residue, which was purified by column chromatography (ethyl ether/*n*-hexane, 1:2) to afford in 55% yield the pure (+)-(*S*)-**2c**, [α]_D_ = +39 (c, 1.28, acetone) (15.6% op); ^1^H-NMR (60 MHz, CDCl_3_) δ 7.75–7.30 (m, 5H, H-Ar); 2.70 (s, 6H, (CH_3_)_2_N)

*Conversion of (+)-(S)–N-p-tolylsulfinylpyrrolidine* (**1d**) *into its enantiomer* (−)-(R)-**1d***.* To a solution of (+)-**1d**, [α]_D_ =+215 (81% op) (0.002 mol) in nitromethane (30 mL) methyl triflate (0.004 mol) was added at room temperature. After 30 min, the solvent and an excess of methyl triflate were evaporated to give the corresponding sulfonium salt [^1^H-NMR (60 MHz, CD_3_NO_2_) δ 730–780 (q, 4H), 4.25 (s, 3H), 3.10–3.90 (m, 4H), 2.39 (s, 3H). The crude sulfonium triflate obtained as above was dissolved immediately in water (30 mL) and a solution of sodium hydroxide (0.02 N) was added, When pH of the reaction solution reached the value above **7** (phenolphthalein assay, red colour) the reaction was terminated. The water solution was washed with *n*-hexane (3 × 40 mL) and extracted with chloroform (1 × 20 mL). The combined organic extracts were dried over MgSO_4_ and evaporated to give the sulfinamide (−)-(*R*)-**1d** as a white solid in 70% yield; [α]_D_ = −175 (c, 1.16 ethanol) (65.8% op), mp. 43–45 °C; ^1^H-NMR (60 MHz, CD_3_NO_2_) δ 7.50. and 7.10 (AB-system, 4H, H-Ar, *J*_AB_ = 8 Hz); 3.41–2.57 (m, 4H, (CH_2_CH_2_)_2_N); 2.27 (s, 3H, CH_3_-Ar); 1.85–1.55 (m, 4H, (CH_2_CH_2_)_2_N).

### 3.3. Optically Active Alkyl p-Toluenesulfinates **5** and Alkyl Benzenesulfinates **6** from Acid Catalyzed Alcoholysis of Optically Active Sulfinamides **1** and **2**

#### General Procedure

To a solution of sulfinamide **1** or **2** (0.001 mol) in an appropriate alcohol (5 mL) was added acidic catalyst (0.002 mol) The reaction progress was followed polarimetrically. When the reaction was completed, water (25 mL) was added and the resulting solution was extracted with *n*-hexane (2 × 20 mL) and chloroform (1 × 20 mL). The combined organic extracts were dried over MgSO_4_. After evaporation of the solvents, sulfinate esters **5** and **6** were purified by column chromatography (silica gel 200–300 mesh, *n*-hexane-ethyl ether, 10:1). Their optical rotations, optical purities and absolute configurations are given in Table 2, Table 3 and Table 4. In Table 11 the selected spectroscopic data of sulfinates **5** and **6** are collected.

**Table 11:** Spectroscopic properties of O-alkyl arenesulfinates **5** and **6** ArS(O)OR’.

No	Ar	R	IR(S=O) cm^−1^	^1^H-NMR (60 MHz, CDCl_3_ δ (ppm)
**5a**	*p*-Tol	Me	1126	7.45 and 7.25 (AB-system, 4H, H-Ar, *J*_AB_ = 7.5 Hz); 3.30 (s, 3H, CH_3_O); 2.35 (s, 3H, CH_3_-Ar).
**5b**	*p*-Tol	Et	1120	7.45 and 7.25 (AB-system, 4H, H-Ar, *J*_AB_ = 8 Hz); 3.90 and 3.50 (ABX_3_-system, 2H, CH_3_-O, *J*_AB_ = 10.5 Hz, *J*_AX_ = *J*_BX_ = 7 Hz); 2.40 (s, 3H, CH_3_-Ar); 1.20 (t, 3H, CH_3_CH_2_, *J* = 7 Hz)
**5c**	*p*-Tol	Pr^n^	1125	7.45 and 7.23 (AB-system, 4H, H-Ar, *J*_AB_ = 8.0 Hz); 3.82 and 3.44 (ABX_2_-system, 2H, CH_2_O, *J*_AB_ = 8.5 Hz, *J*_AX_ = *J*_BX_ = 7 Hz); 1.60 (sx, 2H, CH_3_CH_2_, *J* = 7 Hz); 0.9 (t, 3H, CH_3_CH_2_, *J* = 7Hz)
**5d**	*p*-Tol	Pr^i^	1130	7.50 and 7.30 (AB-syst, 4H, H-Ar, *J*_AB_ = 8 Hz); 4.50 (sp, 1H, (CH_3_)_2_CHO, *J* = 6 Hz); 2,35 (s, 3H, CH_3_-Ar); 1.31 and 1.18 (d,d, 6H, (CH_3_)_2_CH, *J* = 6 Hz)
**5d'**	*p*-Tol	(CD_3_)_2_CH	1130	7.45 and 7.20 (AB-system, 4H, H-Ar, *J*_AB_ = 8.5 Hz); 4.44 (m, 1H, CH(CD_3_)_2_); 2.35 (s,3H, CH_3_-Ar)
**5d''**	*p*-Tol	(CF_3_)_2_CH	–	7.50 and 7.30 (AB-system, 4H, H-Ar. *J*_AB_ = 8.5 Hz) 4.35 (sp, 1H, (CF_3_)_2_CH, *J*_F-H_ = 6 Hz); 2.40 (s, 3H, CH_3_-Ar).^19^F-NMR (CDCl_3_); δ = +95.2, (d, 6F, (CF_3_)CH, *J*_F-H_ = 6Hz) (C_6_F_6_ as internal standard)
**5j**	*p*-Tol	Hex^c^	1130	7.40 and 7.25 (AB-system, 4H, H-Ar, *J*_AB_ = 8 Hz); 4.10–4.35 (m, 1H, CHO); 2.35 (s, 3H, CH_3_-Ar); 2.10–1.05 (m, 10H, Cy-H)
**5k**	*p*-Tol	Pen^c^	1125	7.45 and 7.20 (AB-system, 4H, H-Ar, *J*_AB_ = 8 Hz); 4.60(m, 1H, CHO); 2.40 (s, 3H, CH_3_-Ar);1.95–1.30 (m, 8H, Cp-H)
**5m**	*p*-Tol	Bu^i^	1128	7.60 and 7.35 (AB-system, 4H, H-Ar, *J*_AB_ = 8 Hz); 4.00–3.20 (m, 2H, OCH_2_CH); 2.40 (s, 3H, CH_3_-Ar); 1.85 (m, 1H, (CH_3_)_2_CH); 0.90 (d, 6H, (CH_3_)_2_CH, *J* = 6 Hz)
**5n**	*p*-Tol	Bu^t^	1125	7.45 and 7.20 (AB-system, 4H, H-Ar, *J*_AB_ = 8.5 Hz); 2.35 (s, 3H, CH_3_Ar); 1.45 (s, 9H, (CH_3_)_3_CO)
**5l**	*p*-Tol	Pentyl-3	–	7.70 and 7.35 (AB-system, 4H, H-Ar, *J*_AB_ = 8 Hz); 4.20 (mc, 1H, CHO); 2.35 (s, 3H, CH_3_-Ar); 1.90–1.20 (m, 4H, CH_3_CH_2_-O); 0.95 and 0.90 (t,t, 6H, CH_3_CH_2_CHO, *J* = 6 Hz)
**6a**	Ph	Me	1125	7.60–7.35 (m, 5H, H-Ar), 3.32 (s, 3H, CH_3_O)
**6b**	P	Et	–	7.70–7.40 (m, 5H, H-Ar), 4.00 (q, 2H, CH_3_CH_2_O, *J* = 7 Hz), 1.30 (t, 3H, CH_3_CH_2_, *J* = 7 Hz)

s-singlet, d-doublet, dd-double doublet, d,d-two doublets, t-triplet, t,t-two triplets, q-quartet, sx-sextet, sp-septet, m-multiplet, Pen^c^-cyclopentyl, Hex^c^-cyclohexyl.

### 3.4. Conversion of Selected Sulfinates **5** into Methyl p-tolyl Sulfoxide

#### General Procedure

To a freshly prepared methylmagnesium iodide (0.015 mol) a solution of the appropriate sulfinate (0.0005 mol) in ether (50 mL) was added dropwise. After stirring for 2 h, to the reaction mixture saturated NH_4_Cl aqueous solution (1 × 30 mL) was added. The separated ethereal layer was washed with 3% aqueous solution of HCl (1 × 10 mL) and 3% aqueous solution of NaHCO_4_ (2 × 20 mL). The organic phase was dried over MgSO_4_. Evaporation of the solvent afforded methyl *p*-tolyl sulfoxide which was purified by column chromatography (silica gel, 200–300 mesh, methylene chloride); mp. 71–73 °C; ^1^H-NMR (60 MHz, CDCl_3_) δ 7.55 and 7.35 (AB-system, 4H, H-Ar, *J*_AB_ = 8 Hz), 2.60 (s, 3H, CH_3_-SO), 2.35 (s, 3H, CH_3_-Ar); IR (KBr, cm^−1^) ν 1060, 1080 (S=O). Optical rotations of the sulfoxides obtained from sulfinates **5** and **6** are summarized in Table 12.

**Table 12:** Determination of optical purity of sulfinates (−)-5 by their conversion into (+)-(*R*)-methyl *p*-tolyl sulfoxide.

*p*-Tol S(O)OR				MeS(O)-Tol-*p*		
No	R	[α]_D_	Conf.	[α]_D_	Op (%)	Conf.
**5e**	CH_2_=CH–CH_2_	−106.5	*S*	+108.0	73	*R*
**5f**	CH_2_=CHCH_2_	−86.0	*S*	+111.3	77	*R*
**5d'**	(CD_3_)_2_CH	−19.8	*S*	+13.6	9.2	*R*
**5d''**	(CF_3_)_2_CH	−10.3	*S*	+7.5	5.1	*R*
**5j**	Hex^c^	−23.9	*S*	+20.6	13.8	*R*
**5k**	Pen^c^	−6.5	*S*	+5.2	3.5	*R*
**5l**	Et_2_CH	−11.7	*S*	+9.2	6.2	*R*
**5m**	Bu^i^	−8.2	*S*	+6.2	4.2	*R*
